# Exploring the Role of Pattern Recognition Receptors as Immunostimulatory Molecules

**DOI:** 10.1002/iid3.70150

**Published:** 2025-05-21

**Authors:** Meenal Sharma, Priyanka Wagh, Tanvi Shinde, Diptee Trimbake, Anuradha S. Tripathy

**Affiliations:** ^1^ Department of Dengue and Chikungunya Indian Council of Medical Research‐National Institute of Virology Pune India

**Keywords:** adjuvants, CLRs, DNA sensors, NLRs, PRRs, RLRs, STING, TLRs, vaccines

## Abstract

**Background:**

Pattern recognition receptors (PRRs) are the receptors of the innate immune system that play a vital role in initiating innate immune response. PRRs recognize pathogen associated molecular patterns (PAMPs) and activate immune cells through a signaling cascade. Due to this remarkable ability to recognize pathogenic microbes and elucidation of an immune response in a well‐organized manner, PRR agonizts are likely to have great potential as vaccine adjuvants. Recent advancements in vaccine development raised concerns regarding the reduced immunogenicity of various vaccines, questioning the vaccine efficacy. In such cases, the use of an adjuvant becomes crucial. Understanding the structure and downstream signaling of PRRs will provide the possibility of developing a novel therapeutic approach.

**Method:**

The rapidly evolving field of immunology and vaccinology, coupled with the increasing focus on PRRs in disease therapy, demands a comprehensive overview. In this review, we provide all‐inclusive and contemporary gist on PRRs and the applications of their agonizts. We explored the potential of PRR agonizts as vaccine adjuvant. The current review integrates the basic understanding of PRRs and recent findings highlighting emerging trends of the same.

**Result:**

Our review highlights that combining multiple PRR agonizts could offer synergistic benefits. This approach might prove advantageous and could potentially enhance vaccine efficacy and reduce the need for excessive immunogens.

**Conclusion:**

A comprehensive understanding of PRR subset, agonizts of PRR and their application in vaccine adjuvant. This knowledge will be significant in formulating vaccine approaches.

## Summary

- This mini‐review summarizes different classes of Pattern Recognition Receptors (PRRs) and the role of their agonizts that have been identified.

- The mini‐review has elaborated on the role of PRRs in the development of therapeutic vaccine adjuvants.

## Graphical abstract

This mini‐review summarizes different classes of pattern recognition receptors (PRRs) and the role of their agonizts that have been identified and also outlines the role of PRR ligands as immunostimulatory molecules. Overall, the mini‐review has elaborated on the role of PRRs in the development of therapeutic vaccine adjuvants.

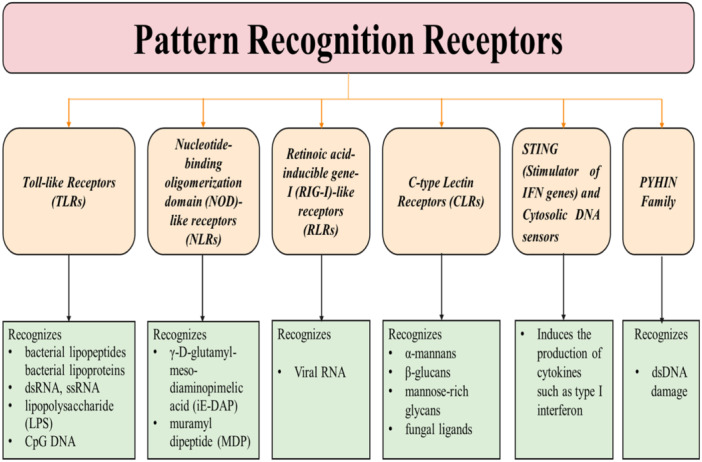

## 1 Introduction

A review article resolves definitional ambiguities and outlines the scope of the topic. A current comprehensive review of pattern recognition receptor (PRR) agonizts in vaccine development is crucial for several reasons: it consolidates existing knowledge, identifies research gaps, and explores practical applications, thereby enhancing vaccine effectiveness. Such reviews guide research directions, influence funding, and serve as valuable educational resources. They also have significant implications for public health policy and clinical practices, helping to optimize vaccine strategies and improve immunization outcomes.

Vaccination is a protective route of mirroring infection in the body resulting in the activation of the immune system to generate an effective immune response. A vaccine is made up of an antigen, a protein or a carbohydrate derived from a pathogen. These antigens initiate the immune response inside the host. Adjuvants are added to the vaccine to enhance the immune response. Live attenuated vaccines, inactivated vaccines, mRNA vaccines, DNA vaccines, recombinant vaccines, multivalent subunit vaccines, polysaccharides, conjugate vaccines, toxoid vaccines and viral vector vaccines are different types of vaccines [1]. Live attenuated vaccines comprise weakened versions of the pathogen, which can replicate inside the host, ensuring long‐lasting immunity. These vaccines are considered highly effective but are associated with a concern of reversion of the pathogen to its virulent form. Measles, mumps, and rubella (MMR) vaccine, varicella (chickenpox) vaccine are widely used live attenuated vaccines [2]. Inactivated vaccines contain inactivated virulent pathogens. These vaccines generally offer a weaker immune response than live attenuated vaccines. To get the expected immune response with inactivated vaccines, several booster doses may be required. Inactivated vaccines are utilized to provide protection against influenza, poliovirus, rabies and hepatitis A virus [3]. The whole virus‐killed vaccines are incompetent of replication due to the viral inactivation steps, including crosslinking or viral splitting. Although, these types of vaccines lack reversion to their virulent form, safety concerns remain [4]. The inactivated toxoid is considered a traditional vaccine which are highly potent. Still, there are safety concerns and issues with the manufacturing process and this conventional approach does not work in some non‐cultivable microorganisms [5]. These limitations led to the introduction of subunit and recombinant protein vaccines, which are highly purified antigens that require only a part of the pathogen to generate a protective immune response. These antigens are safe, cannot revert to a virulent form, and are easier to manufacture, leading to vaccine development improvements. However, several vaccines cannot generate the expected immune responses required to induce the desired immunogenicity against the target antigen. In such cases, the importance of adjuvants become evident. Thus, introducing adjuvants in vaccines is considered helpful in enhancing the immunogenicity of these weaker antigens and improving the overall potency and efficacy of poorly immunogenic subunit vaccines.

### 1.1 Adjuvants

Adjuvants are essential for vaccines to augment the immune response by improving antigen presentation, activating innate immunity, and inducing a more robust and long‐lasting adaptive immune response. Although vaccines have been successfully developed for several human diseases, the process of development of adjuvants has been categorized as the “slowest process.” The word adjuvant is derived from the Latin word “adjuvant,” meaning “to help” [6]. Gaston Ramon, a French veterinarian working at the Pasteur Institute, composed the term “adjuvant“ in 1920. He observed that post vaccination against diphtheria, the horses who developed inflammatory blisters at the site of injection had stronger antibody titers. He later explored the link between these inflammatory abscesses and immune response in terms of antibody titers. He injected breadcrumbs, starch or tapioca, and inactivated toxin, leading to increased anti‐sera production. This confirmed that substances other than antigens of interest can induce antibody production [7]. In 1926, Alexander T. Glenny discovered that precipitated antigen on aluminum potassium sulfate produced better antibody response than soluble antigen alone. This marked the initial clue of aluminum salts as adjuvants. Aluminum adjuvants have been utilized in vaccination since 1932 [8]. Alum was prominently used in tetanus and diphtheria vaccine formulation. Following this, MF59/Fluad, an oil‐in‐water emulsion adjuvant, was used for the influenza vaccine, and AS04 was used for hepatitis B virus (HBV) and human papilomavirus (HPV). AS01 was incorporated in the shingles vaccine Shingrix and the malaria vaccine Mosquirix. AS03 adjuvant was used for the pandemic influenza vaccines Pandemrix and Arepanrix. cytosine phosphoguanosine (CpG) 1018 was utilized in the hepatitis B vaccine Heplisav‐B vaccine [6]. Despite the universal use of adjuvants, the complete mechanism of adjuvant in eliciting immune response is not fully known.

### 1.2 Pattern Recognition Receptor

Receptors of the innate immune system recognize conserved molecular structures, such as pathogen‐associated molecular patterns (PAMPs), which are present in the foreign pathogenic microorganisms and are absent in the host system. These receptors are pattern recognition receptors (PRRs) [9]. PRRs are categorized as soluble extracellular, membrane‐associated and intracellular receptors depending upon their localization, ligand specificity, and their functions. They are classified into five families, including toll like receptors (TLR), retinoic acid inducible gene 1 (RIG‐I)‐like Receptors (RLRs), nucleotide‐binding and oligomerization domain (NOD)‐like receptors (NLRs), C‐type Lectin Receptors and DNA sensors. PRRs are mainly expressed on immune cells such as dendritic cells, macrophages, and B cells [10]. Activating PRRs on host cells promote the production of pro inflammatory factors, chemokines and interferons (INFs). The adjuvant mechanism is incompletely understood and has remained mostly unexplored, however, the way the innate immune system identifies the microbes has been widely studied. This proposes a vast area for exploring the features related to adjuvant design and development. It is believed that PRRs recognize vaccine adjuvants as PAMPs and hence trigger the innate immune response. Owing to the immunostimulatory effect of PRRs, research has shifted towards using PRR agonizts as vaccine adjuvants. Table 1 summarizes the subsets of PRRs in terms of their agonists and commercially available potential adjuvants.

**Table 1:** Summary of PRRs.

Sr. No.	PRR	Location	Function	Description	Agonizts	Commercialized molecules	Reference
1	**Toll‐like receptors (TLRs)**						
1a	TLR1	Plasma membrane	Recognition of bacterial lipopeptides	Recognizes triacylated lipopeptides from Gram‐negative bacteria and diacylated lipopeptides from Mycoplasma spp.	Bacterial lipopeptides	Pam3CSK4	[16]
1b	TLR2	Plasma membrane	Recognition of bacterial lipoproteins	Recognizes, bacterial proteins (e.g., V‐antigen from Yersinia), hemagglutinins from smallpox virus, glycolipids, glycopeptides, and lipoproteins from E. coli, B. burgdorferi, M. tuberculosis	Bacterial lipoproteins, peptidoglycan	SMP‐105	[17]
						CBLB612	[18]
1c	TLR3	Endosomes	Recognition of double‐stranded RNA (dsRNA)	Recognizes viral infectionsEx. West Nile virus, RSV, encephalomyocarditis virus (EMCV)	Double‐stranded RNA (dsRNA)	poly(I:C)	[19]
						poly(ICLC) poly(IC12U)	[20]
						ARNAX	[21]
1d	TLR4	Plasma membrane	Recognition of lipopolysaccharide (LPS)	Recognizes glycosaminophospholipids from Trypanosoma, fusion proteins from RSV, envelope proteins from mouse mammary tumor virus (MMTV)	Lipopolysaccharide (LPS), lipoteichoic acid (LTA)	MPLA	[22]
						FP molecules	[23]
						GLA‐SE	[24]
						AS04	[25]
						OK‐432	[26]
1e	TLR5	Plasma membrane	Recognition of flagellin	Recognizes invasion of microorganisms that cause respiratory tract infections and gastrointestinal infections	Flagellin	CBLB502, M‐VM3	[27]
1f	TLR6	Plasma membrane	Recognition of bacterial lipoproteins	Collaborates with TLR2	Bacterial lipoproteins, zymosan		
1g	TLR7	Endosomes	Recognition of imidazoquinoline compounds	Recognizes viral infections	Imidazoquinoline compounds	TLR7‐852A	[28]
						Gardiquimod	[29]
1h	TLR8	Endosomes	Recognition of single‐stranded RNA (ssRNA)	Recognizes viral infections	ssRNA, imidazoquinoline compounds	Motolimod	[30]
						VTX‐1463	[31]
						R848	[32]
1i	TLR9	Endosomes	Recognition of CpG DNA	Recognizes unmethylated CpG DNA in bacteria and viruses	CpG DNA	CpG 7909 CpG‐1826 CpG 1018 SD‐101 KSK‐CpG MGN1703	[33, 34]

Sr. No.	PRR	Location	Function	Description	Agonist	Reference
2	**Nucleotide‐binding oligomerization domain (NOD)‐like receptors (NLRs)**					
2a	NOD1	Cytoplasm	Recognition of γ‐d‐glutamyl‐meso‐diaminopimelic acid (iE‐DAP)	Recognizes bacterial peptidoglycans	FK565 FK156	[45]
2b	NOD2	Cytoplasm	Recognition of muramyl dipeptide (MDP)	Recognizes bacterial peptidoglycans	MDP	[41, 45]
3	**Retinoic acid‐inducible gene‐I (RIG‐I)‐like receptors (RLRs)**					
3a	RIG‐I	Cytoplasm	Recognition of viral RNA	Recognizes viral infections		[36]
3b	MDA5					
3c	LGP2					
4	**C‐type lectin receptors (CLRs)**				Trehalose dibehenate	[48, 49]
4a	Dectin‐1	Plasma membrane	Recognition of β‐glucans	Recognizes fungal cell wall components		
4b	Dectin‐2	Plasma membrane	Recognition of α‐mannans	Recognizes fungal cell wall components		
4c	Mannose receptor (CD206)	Plasma membrane	Recognition of mannose‐rich glycans	Involved in endocytosis and antigen presentation		
4d	DC‐SIGN (CD209)	Plasma membrane	Recognition of high‐mannose glycans	Expressed on dendritic cells and macrophages		
4e	Macrophage‐inducible C‐type lectin (Mincle)	Plasma membrane	Recognition of fungal ligands	Involved in antifungal immune responses		

### 1.3 PRRs and Ligand‐Recognition Mechanisms

#### 1.3.1 Toll‐Like Receptors (TLRs)

The TLR family was the first group of PRRs to be characterized. The Toll gene was first discovered in Drosophila. Due to the similarity of the cell surface receptors to the toll protein they are named as Toll Like Receptors. So far, 222 TLRs have been characterized in invertebrates and 28 TLRs in vertebrates. Among these, 13 TLRs are found in mammals and humans possess only 10 [11]. All innate immune cells, i.e. dendritic cells (DCs), natural killer (NK) cells, macrophages, neutrophils, mast cells, eosinophils and basophils, express TLRs [12]. TLRs are classified based on their location into membrane‐bound or intracellular endosome TLRs. TLR1, 2, 4, 5, 6, and 10 are cell membrane TLRs, while TLR3, 7, 8, and 9 are intracellular TLRs [13]. TLRs have three structural domains, that is, the N terminal domain located on the surface of the membrane for recognition of PAMPs and ligand binding. A transmembrane domain and C terminal cytoplasmic intracellular domain for downstream signaling [14]. TLRs activate two pathways depending upon stimulation with a particular microbial ligand. One is the MyD88‐dependent pathway leading to the production of inflammatory cytokines. Apart from TLR3, all TLRs utilize this pathway. The second pathway is TRIF‐dependent, associated with the stimulation of interferon type‐1 and is used by TLR3 and 4 [15]. The signaling cascade depends upon the ligand interacting TLR type, that carries a leucine‐rich repeat (LRR) segment. It recognizes PAMP/DAMP and downstream signaling molecules leading to an inflammatory response. This makes TLRs an excellent target for adjuvants to induce immune response effectively.

TLR1/2 forms a heterodimer recognizing various bacterial lipid structures and cell wall components like triacylated lipoproteins, lipoteichoic acid, and β‐glucans. Pam3CSK4 (Pam3‐Cys‐Ser‐Lys4) is a synthetic bacterial lipopeptide and is a well‐characterized potential activator of TLR1/2. It is proved that Pam3CSK4 enhances the binding of respiratory syncytial virus. to its target cell, eventually initiating a robust immune response [16]. SMP105 is a TLR2 agonist isolated from *M. bovis* BCG Tokyo. It is proven to enhance the immune response by activating the nuclear factor‐kB promoter and eventually enhancing several interferon‐γ producing cells and CTLs [17]. CBLB612, ISA201 and OPN305 are synthetic ligands for TLR2. These agonizts are in the phase 2 clinical trial for cancer therapy [18]. Poly (I:C), the TLR3 agonist not only activates the immune system but also offers apoptosis of cancer cells. Studies have shown that the combination of IFN‐ α along with poly (I:C) is the most effective strategy for apoptosis of cancerous cells [19]. Studies focusing on the use of poly(IC:LC), a synthetic derivative of poly(I:C), showed that poly(IC:LC), along with antigen‐pulsed DCs, could boost antitumour immunity, i.e. expand tumor‐reactive T cells in patients with pancreatic cancer. Modified version of poly(I:C)‐ poly(IC_12_U), induces the production of 2′,5′‐OAS, which serves as an antiviral effector [20]. ARNAX, another TLR3 agonist enhances memory CD8 + T cells and rejects tumor re implantation [21]. TLR4 agonist 3‐O‐desacyl‐4′‐monophosphoryl lipid A (MPLA) effectively cleared bacterial infection with the help of myeloid cells with effective phagocytic functions [22]. FP molecules, synthetic monosaccharide‐based TLR4 agonizts, bind to the TLR4/MD‐2 dimer with high affinity, stabilize its active conformation. This binding activate MyD88‐ and TRIF‐dependent signaling pathways and the NLRP3 inflammasome. This exhibits adjuvant activity with antibody response with potency comparable to MPLA, while showing no in vivo toxicity [23]. Research on improved tuberculosis vaccines used GLA‐SE adjuvant and TLR4 agonist, which proved to induce robust T‐helper cellular immune response [24]. Adjuvant System 04 (AS04) is a well‐known TLR4 agonist‐based adjuvant licensed for use in human vaccines. It incorporates MPLA and aluminum salt [25]. OK‐432 is yet another synthetic TLR4 agonist which is proven to have immunostimulatory effects [26]. A derivative of flagellin, CBLB502 is a TLR5 stimulant being developed for anticancer applications [27]. Mobilan‐VM3 is a secreted version of TLR5 ligand is demonstrated to be useful not only as a therapy, but also as a prophylactic anticancer vaccine [27]. 852A, the TLR7 agonist prevents in vitro proliferation of some tumor cells (Hs294T and 769‐P) and delays tumor growth in vivo in a DCs and interferon‐α‐dependent manner [28]. Another TLR7 immune system modifier, gardiquimod, serves as a reverse transcriptase inhibitor, offering a promising path for therapeutic development against HIV‐1 [29]. Motolimod, also known as VTX‐2337, is a TLR8 activator. It initiates the immune response with the help of effectors immune cells such as NK cells, DCs, and monocytes [30]. Another TLR 8 agonist VTX‐1463, an intranasal formulation, is in Phase I clinical evaluation for allergic rhinitis. VTX‐763, a member of the VTX family, is in preclinical studies for its potential to decrease the severity of autoimmune inflammation. It is also demonstrated to activate both neonatal and adult leukocytes [31]. R848, one more TLR8 agonist provides antitumor properties in experimental models of breast cancer [32]. It has been demonstrated that CpG oligodeoxynucleotides stimulate TLR9. This causes B cells to secret interleukin‐6. Research proved that a low dose of radiation along with CpG‐C ODN leads to reduced T regulatory cell counts and induction of tumor‐specific CD8+ and CD4+ effector T‐cells [33]. In preclinical studies on a DNA‐based TLR9 agonist, MGN1703 shown promise in anticancer therapy [34].

#### 1.3.2 Retinoic Acid‐Inducible Gene 1 (RIG‐I)‐Like Receptors (RLRs)

TLRs can recognize viruses present on the cell surface or those which are in the endosomal compartment of the host cell. However, viruses usually replicate inside the cell. Hence effective intracellular receptors are required for an effective antiviral response. RIG‐I recognizes viral RNA and is specific to RNA viruses. RIG‐1 does not bind to viral dsDNA except for Epstein‐Barr virus (EBV). RIG‐I was first discovered in the cells of acute promyelocytic leukemia induced by retinoic acid [35]. RIG‐like receptors (RLRs) comprises of RIG‐I, melanoma differentiation‐associated gene 5 (MDA5), and laboratory of Genetics and Physiology 2 (LGP2) [36]. The structures of all three RLRs are similar, consisting of a C‐terminal domain called the repressor domain (RD) and a DExD/H‐box helicase domain. RIG‐I and MDA5 have caspase activation and recruitment domains (CARDs), which carry out downstream signaling cascades. LGP2 lacks a CARD domain. The CARDs engage with the CARD of the protein known as the mitochondrial activator of viral signaling (MAVS), a crucial signaling adapter protein for RLRs. The signaling cascade leads to the transcription of various innate immune response genes, including IFNs, direct antiviral genes, and pro inflammatory genes [37]. The expression of RLR is usually low in resting cells, but it increases after exposure to a viral infection or IFN. RLR signaling is enhanced by ssRNA PAMPs as observed in influenza virus, Ebola virus and other RNA viruses by poly‐uridine‐rich RNA motifs [38]. RIG‐I in the cell primarily recognizes influenza, vesicular stomatitis, Sendai, and Japanese encephalitis viruses. MDA5 mainly detects short RNA viruses like poliovirus. MDA5 also produces polycytidylic acid (poly I:C), the dsRNA analog [36]. Separate but overlapping groups of viruses activate RIG‐I and MDA5. Although they share similar structure and function, they identify different RNA ligands. RIG‐I strongly recognizes ligands such as blunt‐ended dsRNAs of at least 10 bp with a 5^|^ triphosphate group. RIG‐I is also activated by long dsRNA molecules that are more than 200 bp long, regardless of the 5^|^ end, as well as 30‐monophosphorylated ssRNAs and poly U/UC‐rich stretches of ssRNA. Certain sections of negative sense viral RNAs can activate MDA5, which can detect lengthy dsRNA molecules, preferably with higher‐order RNA structures. AU‐rich ssRNAs are likewise preferred by both RLRs [38].

#### 1.3.3 Nucleotide‐Binding and Oligomerization Domain NLRs

Nucleotide‐binding oligomerization domain (NOD)‐like receptors (NLRs) are a family of intracellular PRR in the cytosol. These receptors are essential in recognizing PAMPs associated with bacterial origin [39]. NLRs were first discovered in plants as resistance (R) genes. These R genes play a vital role in plant defense against microbial and parasitic pathogens [40]. To date 23 NLR family members are discovered in human genome [40]. NLRs are predominantly expressed on phagocytes. They are also expressed on macrophages and neutrophils. NLR proteins have central NOD NACHT that is, NLR apoptosis inhibitory protein (NAIP), Class II Transactivator (CIITA), heterokaryon incompatibility protein (HET), telomerase‐associated protein 2 (TP‐2), N‐terminal effector domain, and C‐terminal LRRs. Oligomerization and dNTPase activity are the two essential roles of NACHT, while the C‐terminal LRR domain is involved in ligand binding and activator sensing. N‐terminal domain interacts with other proteins and performs effector functions. Based on the N‐terminal domain, the NLRs are classified into four sub‐families: the baculoviral inhibitory repeat‐like domain (NLRB), the acidic transactivation domain (NLRA), the caspase activation and recruitment domain (CARD; NLRC) and the pyrin domain (NLRP). Most NLRs act as PRRs and detect various ligands from the host cells (ATPs, uric acid etc.), microbial components (peptidoglycan, flagellin), while some instead respond to cytokines such as interferons. The activated NLRs are also broadly divided into four functional categories: inflammasome assembly involved in activation of caspase‐1(results in processing and maturation of pro‐inflammatory cytokine), signaling transduction, transcription activation and autophagy. NOD1 detects d‐glutamyl‐meso‐diaminopimelic acid (iE‐DAP), a peptidoglycan component that is found only in Gram‐negative bacteria, and NOD2 recognizes muramyl dipeptide (MDP) from both gram negative and gram‐positive bacteria [41]. Stimulation of specific NLRs such as NOD1 and NOD2 triggers the activation of a signaling cascade, which activates NF‐κB and MAPKs. These pathways eventually lead to the transcription of various genes involved in both innate and adaptive immune responses [42]. Research demonstrates that, genetic variations within NLR genes have been linked with immune dysregulation, contributing to the development or increased susceptibility to various inflammatory diseases [43]. NOD1 and NOD2 agonizts can synergize with ligands for TLRs. This synergy leads to the enhanced production of pro inflammatory cytokines and antimicrobial molecules. Additionally, NOD1 stimulation has been shown to drive Th2‐dependent antigen‐specific adaptive immunity. This suggests that NLRs can influence the development of different T helper cell subsets (Th1, Th2, Th17) involved in adaptive immune responses, potentially in cooperation with TLR signaling [44]. NOD1 agonizts FK565, FK156 and NOD2 agonist MDP mimic the peptidoglycan layer of bacteria. These agonizts are proven to be potent adjuvants that can trigger cell‐mediated immunity. These agonizts and TLR3, TLR4, and TLR9 immunostimulatory molecules induce DCs to produce IL‐12 and IFN‐γ and eventually promote Th1‐lineage immune responses [45].

#### 1.3.4 C‐Type Lectin Receptors (CLRs)

CLRs are a subfamily of PRRs that mainly recognize mannose, fucose and glucan carbohydrate structures. They are expressed in immune cells such as macrophages, DCs, monocytes, and Langerhans cells (LCs) [46]. CLRs have one or more carbohydrate recognition domains (CRD) that can bind to carbohydrates, proteins, lipid molecules and inorganic compounds in a Ca2 + ‐dependent or Ca2 + ‐independent manner. CLRs are classified as type I transmembrane proteins with multiple carbohydrate recognition domain (CRDs) and type II transmembrane proteins with a single CRD [47]. Important subclasses of CLR include Langerin, DC‐SIGN, Dectin‐1, Dectin‐2, DCIR, DCAR, and BDCA‐2 [48]. These are present on antigen presenting cells, which recognize various PAMPs and play crucial roles in shaping immune responses. These CLRs bind to specific sugars on antigens and trigger the signaling cascade, like uptake, presentation to T cells, cytokine production, and immune modulation. These processes contribute to the intricate interplay between the immune system and infectious agents. CLRs trigger an immune response through two different pathways: (a) immunoreceptor tyrosine‐based activation motifs (ITAMs), which induce the activation pathway and (b) myeloid inhibitory C‐type lectin (MICL) receptor pathway, which suppresses the inflammatory pathways induced by the activating receptors [48]. CLR can enhance vaccine efficacy by using synthetic agonizts like macrophage‐inducible C‐type lectin (Mincle) agonist trehalose dibehenate (tDB). Trehalose dibehenate is a synthetic CLR ligand proven to activate new‐born human DCs in vitro [49].

#### 1.3.5 Stimulator of IFN Genes (STING) and Cytosolic DNA Sensors

The STING is an intracellular PRR present in the endoplasmic reticulum. It induces the production of cytokines such as type I interferon [50]. The cyclic GMP‐AMP synthase (cGAS‐STING) signaling pathway initiates with the entrance of pathogenic DNA into the host cytoplasm through infection. GCS catalyzes the synthesis of cGAMP, which binds to STING. STING eventually recruits IκB Kinase (IKK) and TANK‐binding Kinase 1 (TBK1). IKK and TBK1 phosphorylates Interferon Regulatory Factor 3 (IRF3) which along with other transcription factors induce the expression of interferons and inflammatory cytokines such as tumor necrosis factor (TNF), IL‐1b and IL‐6 [51]. STING agonizts are predominantly involved in tumor‐targeted therapeutics. DMXAA, a STING agonist, proven to reduce and regress tumor growth [52]. ADU‐S100, another agonist induce the local production of antiangiogenic factors [53]. MK‐2118, SB11285, GSK3745417, BMS‐986301, BI‐STING (BI 1387446), E7766, TAK‐676, SNX281, SYNB1891, BMS‐986301 are the STING agonizts in clinical development against different types of tumor and anticancer therapies [54].

#### 1.3.6 PYHIN Family

The PYHIN family consists of DNA sensors activated by pathogenic infections and cellular stress conditions, such as DNA damage or breaks. These sensors play a crucial role in the innate immune system by detecting cellular damage. In humans, key members of this family include Absent in Melanoma 2 (AIM2), Interferon Gamma Inducible Protein 16 (IFI16), and Myeloid Cell Nuclear Differentiation Antigen (MNDA). AIM2 and IFI16 are well‐known for detecting viral or abnormal dsDNA, whereas MNDA regulates immune responses in the myeloid cells [55].

AIM2 is found to be active in assembling inflammasome in response to pathogens such as *Francisella tularensis*, *Listeria monocytogenes*, and *Staphylococcus aureus*. AIM2 is also linked to various conditions, including lupus, psoriasis, cervical cancer and cancers of colon and prostate. It senses dsDNA from viruses such as HPV, HBV and EBV contributing to immune responses. AIM2's molecular mechanism involves binding to dsDNA via its HIN domain, triggering inflammasome assembly with Apoptosis‐Associated Speck‐like Protein containing a CARD and pro‐caspase‐1, leading to inflammation. Additionally, AIM2 can promote T regulatory cell stability during inflammation and has been implicated in atherosclerosis progression [55]. IFI16 is a protein involved in the cell cycle regulation and transcriptional control. It contains two hematopoietic interferon‐inducible nuclear (HIN) domains (HINa and HINb) interacting with transcription factors Sp1 and p53, and inhibits the oncogenes such as c‐MYC and RAS, eventually supressing the tumor growth. IFI16 plays a critical role in innate immunity by sensing dsDNA from viruses like Herpes Simplex Virus Type 1, Kaposi's Sarcoma‐Associated Herpes virus, and Human Immunodeficiency Virus (HIV) as well as ssDNA from the damaged cells. Upon detecting viral DNA, IFI16 forms inflammasomes and activates immune pathways, such as the STING/TBK1 pathway, leading to interferon production [56].

## 2 Discussion

PRRs play a role in the host's innate immune system in identifying potential pathogenic microbes. It is important to note that the PRRs do not function in isolation but orchestrate the overall immune response together. Most of the previous studies were focused on the agonizts that bind to particular PRR and the signaling cascade activated by that PRR. However, it is noteworthy that a pathogen might simultaneously show various ligands for different types of PRRs, triggering a complex immune mechanism. Understanding these fundamental host‐pathogen interactions and the cross‐talk mechanism of PRRs might prove helpful in designing vaccine strategies that will induce a robust immune response. In most of the published research articles, research orients mainly around TLRs. It is considerable that TLRs are the first class of PRRs to be discovered and have specificity for many types of PAMPs. The research focusing on the synergistic action of TLR ligands and the rest of the PRR agonizts in vaccine formulation might prove a practical approach to tackle pathogens showcasing multiple PAMPS. It seems reasonable to believe that adjuvants used in combination targeting multiple PRRs may prove useful tool in designing effective vaccines. PRRs have immunostimulatory properties, by which PRR can induce a strong and long‐lasting immune response. Hence, PRRs can be used in therapeutics. PRR agonizts are proven to induce cell death in the tumor microenvironment. Hence, cancer therapeutics with PRRs as vaccine adjuvants or tumor‐targeted drugs hold a promising strategy against cancer. Advancements can be done using PRRs with other combinational therapies.

## 3 Conclusion

The progress in studying the interplay between PRRs and ligands is considerable. PRR agonizts can stimulate potent innate immune responses and hence are essential as adjuvant candidates. The current review is an analysis of different subsets of PRRs, their agonizts as vaccine adjuvants and has summarized on how these molecules activate the immune system. The review has also explored their applications in various vaccine platforms that will broaden our understanding of PRR adjuvants and may pave the way for more precise therapeutic interventions.

## Author Contributions

Conceptualization: Anuradha S. Tripathy. Writing original draft preparation: Meenal Sharma, Priyanka Wagh, Tanvi Shinde, Anuradha S. Tripathy, and Diptee Trimbake. All authors have read and agreed to the final version of the manuscript.

## Conflicts of Interest

The authors declare no conflicts of interest.

## Data Availability

The authors have nothing to report.
